# Point-of-Care-Tests für sexuell übertragbare Infektionen – was ist der aktuelle Stand?

**DOI:** 10.1007/s00103-025-04067-9

**Published:** 2025-06-05

**Authors:** Viviane Bremer, Heinrich Scheiblauer, Maximilian Muenchhoff, Christian Schüttler, Jörg Timm, Michael Baier, Susanne Buder, Kristin Meyer-Schlinkmann, Thomas Neiße, Roger Dumke, Thomas Meyer, Gyde Steffen, Klaus Jansen, Norbert Brockmeyer

**Affiliations:** 1 Abt. für Infektionsepidemiologie, Robert Koch-Institut, Seestr. 10, 13353 Berlin, Deutschland; 2 Prüflabor für In-vitro-Diagnostika am Paul-Ehrlich-Institut (PEI-IVD), Langen, Deutschland; 3 Nationales Referenzzentrum für Retroviren, Max von Pettenkofer-Institut, Ludwig-Maximilians Universität, München, Deutschland; 4 Nationales Referenzzentrum für Hepatitis-B- und -D-Viren, Justus-Liebig-Universität, Gießen, Deutschland; 5 Nationales Referenzzentrum für Hepatitis-B-Viren, Universitätsklinikum Düsseldorf, Heinrich-Heine-Universität, Düsseldorf, Deutschland; 6 Konsiliarlabor für Chlamydien, Institut für Medizinische Mikrobiologie, Universitätsklinikum Jena, Jena, Deutschland; 7 Konsiliarlabor für Gonokokken, Abt. für Infektionskrankheiten, Robert Koch-Institut, Berlin, Deutschland; 8 Konsiliarlabor für Treponema pallidum, MVZ Labor Krone, Bad Salzuflen, Deutschland; 9 Konsiliarlabor für Mykoplasmen, Institut für Medizinische Mikrobiologie und Virologie, Universitätsklinikum Carl Gustav Carus, Technische Universität Dresden, Dresden, Deutschland; 10 Klinik für Dermatologie, Venerologie und Allergologie, Katholisches Klinikum, Ruhr-Universität, Bochum, Deutschland; 11 Deutsche STI-Gesellschaft, Ruhr-Universität, Bochum, Deutschland

**Keywords:** Point-of-Care-Test, Selbsttest, STI, HIV, Hepatitis, Point-of-care test, Self-test, STI, HIV, Hepatitis

## Abstract

Point-of-Care-Tests (PoCT) ermöglichen eine schnelle Diagnostik von sexuell übertragbaren Infektionen (STI) direkt am Einsatzort und spielen eine zentrale Rolle bei der Erreichung der Ziele der Weltgesundheitsorganisation (WHO) zur globalen Eliminierung von Aids und Hepatitis sowie zur Reduzierung von STI bis 2030.

Die neue Verordnung der Europäischen Union für *In-vitro*-Diagnostika erhöht die Qualitätsstandards für PoCT. Alle STI-Tests müssen nun einer strengeren Überprüfung unterzogen werden. Fortschritte in der PoCT-Entwicklung haben die Testmöglichkeiten für HIV, Syphilis und Hepatitis C verbessert, insbesondere durch präqualifizierte Tests der WHO. Die in Deutschland verfügbaren HIV-Selbsttests detektieren jedoch nur die Antikörperkomponente, sodass ein negatives Ergebnis erst 12 Wochen nach Exposition sicher aussagekräftig ist. Für Syphilis gibt es PoCT für das Antikörperscreening, die jedoch im Labor einer Bestätigung bedürfen. Für Chlamydien‑, Gonokokken- und Mykoplasmeninfektionen ist die PoCT-Qualität größtenteils noch unzureichend. Weiterentwicklungen im Bereich der Nukleinsäureamplifikationstests (NAT) zeigen diesbezüglich vielversprechende Ansätze.

PoCT sind besonders vorteilhaft für niedrigschwellige Angebote, die sich an marginalisierte Gruppen richten, da sie eine zeitnahe Diagnosestellung und Behandlung im Rahmen des Testsettings ermöglichen. Herausforderungen bleiben jedoch die Qualitätssicherung und mögliche psychologische Belastungen durch falsch-positive Ergebnisse. Es besteht weiterhin die Notwendigkeit einer kontinuierlichen Weiterentwicklung und Integration von PoCT in das Gesundheitssystem, um den Zugang zur Diagnostik zu verbessern und die Verbreitung von Infektionen zu reduzieren.

## Abstract (en)

Point-of-care tests (PoCTs) enable the rapid diagnosis of sexually transmitted infections (STIs) directly at the point of care and play a central role in achieving the goals of the World Health Organization (WHO) for the global elimination of AIDS and hepatitis and the reduction of STIs by 2030.

The European Union’s new regulation for in vitro diagnostics raises the quality standards for PoCTs. All STI tests must now undergo more rigorous reviews. Advances in PoCT development have improved testing options for HIV, syphilis, and hepatitis C, particularly through WHO prequalified tests. However, the HIV self-tests available in Germany only detect the antibody component, which means that a negative result is only reliably conclusive 12 weeks after exposure. For syphilis, PoCTs are available for antibody screening, but these still require subsequent laboratory confirmation. For infections with chlamydia, gonococci, and mycoplasma, the quality of PoCTs is still largely inadequate. Further developments in the field of nucleic acid amplification tests (NATs) show promising approaches.

PoCTs are particularly beneficial for low-threshold services aimed at marginalized groups, as they enable swift diagnosis and timely treatment within the test setting. However, challenges remain, including quality assurance and potential psychological stress due to false-positive results. There remains a need for continuous development and the integration of PoCTs into the healthcare system in order to improve access to diagnostics and to reduce the spread of infections.

## Hintergrund

Sexuell übertragbare Infektionen (STI), inklusive Infektionen mit dem humanen Immundefizienzvirus (HIV) sowie Hepatitis-B-Virus (HBV) und Hepatitis-C-Virus (HCV), können zu einer hohen Morbidität und Mortalität führen. Für das Jahr 2023 schätzte das Gemeinsame Programm der Vereinten Nationen für HIV/AIDS (UNAIDS) die Anzahl neuer HIV-Infektionen weltweit auf 1,3 Mio. [1]. Im Jahr 2022 lag nach Schätzungen der Weltgesundheitsorganisation (WHO) die Anzahl der HBV- und HCV-Neuinfektionen weltweit bei 1,2 Mio. bzw. 1,0 Mio. und die Anzahl der Todesfälle durch HBV- bzw. HCV-bedingte Spätfolgen wie Leberzirrhose oder hepatozelluläres Karzinom bei 1,1 Mio. bzw. 242.000 [2, 3]. Für das Jahr 2020 wurden durch die WHO weltweit 376 Mio. neue STI-Infektionen bei 15- bis 49-Jährigen geschätzt. Davon entfielen 129 Mio. Fälle auf Infektionen mit *Chlamydia trachomatis* (CT), 82 Mio. auf *Neisseria gonorrhoeae* (NG) und 7,1 Mio. auf Syphilis [4].

In Deutschland wurden für das Jahr 2023 2200 neue HIV-Infektionen geschätzt. Der Anteil der Menschen in Deutschland mit einer nichtdiagnostizierten HIV-Infektion wurde Ende 2023 auf etwa 8200 Personen geschätzt [5]. Im Jahr 2023 wurden dem Robert Koch-Institut (RKI) zudem 22.875 Fälle von HBV, 10.512 Fälle von HCV und 9128 Syphilisinfektionen übermittelt (www.survstat.de).

Die globale Strategie der WHO für HIV, Hepatitis und STI sieht vor, bis 2030 Aids und Hepatitis zu eliminieren und STI-Infektionen substanziell abzusenken. Dies sollte unter anderem durch den Auf- und Ausbau bedarfsgerechter Angebote, inklusive niedrigschwelliger Testmöglichkeiten, erreicht werden [6]. Darauf zielt auch die im Jahr 2016 verabschiedete „Strategie zur Eindämmung von HIV, Hepatitis B und C und anderen sexuell übertragbaren Infektionen“ (BIS 2030) der Bundesregierung ab [7].

Ein wichtiger Baustein für niedrigschwellige Testangebote sind sogenannte Point-of-Care-Tests (PoCT) oder Schnelltests. Diese Tests zeichnen sich dadurch aus, dass das Testergebnis bereits innerhalb weniger Minuten bis zu 2 Stunden nach der Probenentnahme laborunabhängig in der Einrichtung selbst, also am „Point of Care (PoC)“ erhältlich ist. Im März 2020 wurde durch die Änderung des § 24 Infektionsschutzgesetz (IfSG) der Arztvorbehalt für die Testung von HIV, Syphilis und HCV aufgehoben [8]. Bei diesen PoCT ist somit auch eine Nutzung außerhalb medizinischer Einrichtungen als sogenannter Selbsttest ohne Beratung vor und nach dem Test vorgesehen.

In dieser Arbeit werden Vor- und Nachteile der PoCT und regulatorische Aspekte und Meldepflichten bezüglich PoCT dargestellt sowie der aktuelle Stand zu PoCT für den Nachweis von HIV, HCV, Syphilis, Gonokokken, Chlamydien und Mykoplasmen.

## Vor- und Nachteile von PoCT

Mittels PoCT können Personen innerhalb eines einzigen Termins eine Diagnose erhalten und frühzeitig einer gezielten Therapie zugeführt werden. Dadurch kann das Risiko einer Übertragung der Infektion auf andere Personen verringert werden. PoCT bieten für Settings mit vulnerablen oder sehr mobilen Personen Vorteile, da diese oft ihr Testergebnis nicht abholen und somit keine Therapie erhalten [9–12].

Das Prinzip der PoCT basiert meist auf der Lateral-Flow-Technologie (Lateral-Flow-Assay; LFA), eine Technik, die ebenfalls für die PoCT auf Severe Acute Respiratory Syndrome Coronavirus Type 2 (SARS-CoV-2) zum Einsatz kam [13]. Es werden hierbei in wenigen Arbeitsschritten visuell qualitative Ergebnisse erzielt. Diese Verfahren sind einfach in der Handhabung, robust gegen Umwelteinflüsse und können auch von nichtmedizinischem Personal angewandt werden. Insofern können PoCT auch in Einrichtungen angewandt werden, die keine ärztliche oder keine durchgängige Versorgung haben, wie etwa niedrigschwellige Drogenberatungsstellen oder HIV/STI-Beratungsstellen. Für antigenbasierte PoCT zum Nachweis von STI wurden 2021 durch die WHO neue Produktprofile zur Anwendung im Labor oder in einer medizinischen Versorgungseinrichtung definiert [14]. Die Medizinprodukte-Abgabeverordnung und das IfSG regeln, ob die Voraussetzungen für den Gebrauch durch Laien (z. B. Gebrauchstauglichkeit, Verständlichkeit, Ablesbarkeit) erfüllt sind. Ist das der Fall, können diese Tests für die Eigenanwendung genutzt werden. So sind seit Oktober 2018 HIV-Selbsttests im Handel erhältlich. PoCT, sei es bei einer Anwendung in niedrigschwelligen Einrichtungen oder zu Hause, können jedoch eine vollständige Labortestung nicht ersetzen. Ein reaktiver PoCT muss in der Regel im Labor durch Antigentests oder Nukleinsäureamplifikationstests (NAT) bestätigt werden.

Eine weitere Alternative sind NAT, die vor Ort ausgewertet werden (PoC-NAT). Einfache Probenverarbeitung mit wenigen Arbeitsschritten sowie die Standardisierung und Automatisierung mithilfe von konfektionierten Testkits erfordern kein geschultes Laborpersonal zur Durchführung der Diagnostik. Aufgrund dieser Vorteile und durch die Entwicklung von kleinen oder mobilen diagnostischen Geräten mit kurzer Prozessierungsdauer kann die NAT-Diagnostik als PoCT genutzt werden [15]. Zudem kann NAT als Multiplexdiagnostik eingesetzt werden, was den Nachweis mehrerer Pathogene aus derselben Probe und in einem einzigen Testlauf ermöglicht [16, 17]. Die derzeit auf dem Markt befindlichen Tests haben eine Laufzeit von 30–90 min, benötigen eine mobile Diagnoseeinheit mit Strom, sind relativ teuer und erfüllen daher nicht die für PoCT definierten REASSURED-Kriterien (Real-time Connectivity, Ease of Specimen Collection, Affordable, Sensitive, Specific, User-friendly, Rapid and Robust, Equipment-free and Deliverable; [18]). Sie werden als patientennaher („near-patient“) Test und nicht als PoCT bezeichnet.

## Regulatorische Übersicht

Die CE-Zertifizierung und Überwachung von *In-vitro*-Diagnostika (IVD) wird seit dem 26.05.2022 durch die IVD-Verordnung 2017/746 (IVD-VO; [19]) geregelt. Im Hinblick auf Tests zum Nachweis von STI wird damit erwartet, dass bisher unter altem Recht (IVD-Richtlinie 98/79/EG; [20]) von Herstellern selbst deklarierte Tests (z. B. für Syphilis, NG, HPV, HSV) nun als Klasse C unter Beteiligung von benannten Stellen stringenter bewertet werden. Für STI-Tests in der höchsten Risikoklasse D (HIV, HBV, HCV) wird das bestehende Sicherheitsniveau beibehalten und erweitert. Zusätzlich zu gemeinsamen Spezifikationen [21], die Anforderungen an die diagnostische Erprobung der Tests beinhalten, muss ein Referenzlabor der Europäischen Union (EU) einbezogen werden, zu denen das Paul-Ehrlich-Institut (PEI) als eines von insgesamt 5 bisher benannten Laboratorien gehört [22]. Das PEI-IVD-Prüflabor hat entsprechende Erfahrung in der Chargenprüfung und der Evaluierung der Sensitivität und des diagnostischen Fensters in der frühen Infektionsphase von patientennahen (PoCT-)Tests für HIV, HBsAg (ein Oberflächenprotein in der Virushülle von HBV) und HCV im Vergleich mit laborgestützten Tests.

Die IVD-VO beinhaltet auch Änderungen für Inhousetests, v. a. den Nachweis der Gleichwertigkeit mit kommerziellen Produkten, zum Qualitätsmanagementsystem und zur Dokumentation der Herstellung. Die vollständige Umsetzung dieser Anforderungen verzögert sich jedoch bis 2030, wenn die Europäische Datenbank für Medizinprodukte (EUDAMED) zur Recherche von kommerziellen Tests voraussichtlich voll einsatzfähig sein wird. Eine Neuerung der IVD-VO betrifft den sogenannten Fernabsatz, bei dem Testanbieter Probengefäße (z. B. Trockenblutspots) zur Selbstentnahme oder Tests zur Eigenanwendung versenden und Proben oder Testergebnisse in einem Zentrallabor untersucht bzw. ausgewertet und überwacht werden. Der Testanbieter ist dann dafür verantwortlich, dass die IVD-VO zu Inhousetests und CE-Kennzeichnung eingehalten wird.

## HIV

Die Diagnose einer HIV-Infektion beruht auf dem Nachweis von gegen HIV gerichteten Antikörpern sowie gegebenenfalls bestimmter viraler Proteine oder der viralen Nukleinsäure. Die aktuellen WHO-Leitlinien empfehlen ein 2‑stufiges Verfahren mit dem Einsatz von 2 unterschiedlichen HIV-Schnelltests [23]. Diese Empfehlung ist insbesondere in Ländern mit begrenzten Laborkapazitäten praktikabel, wohingegen in Deutschland eine Bestätigung bzw. Spezifitätsüberprüfung positiver HIV-Suchtests weiterhin durch Immunoblot oder NAT sinnvoll ist [24].

Diagnostische Testsysteme, die die EU-Anforderungen an Sicherheit und Leistungsfähigkeit erfüllen, werden mit der CE-Kennzeichnung versehen. Online werden auch HIV-Selbsttests ohne CE-Kennzeichnung angeboten, obwohl sie nicht eingesetzt werden dürfen, da sie nicht verkehrsfähig sind. Zudem ist deren Qualität nicht sichergestellt, da sie nicht der vorgesehenen Bewertung durch eine benannte Stelle und der Chargenprüfung durch ein Prüflabor unterliegen. In Tab. 1 sind die 6 derzeit nach Angaben des Paul-Ehrlich-Instituts auf dem deutschen Markt verfügbaren HIV-Selbsttests mit CE-Kennzeichnung aufgelistet [25]. All diese Tests wurden systematisch in klinischen Studien evaluiert, wobei eine Sensitivität von 96,2–100 % und eine Spezifität von 98,2–100 % beschrieben wurden [26].

**Tab. 1:** Übersicht über verfügbare Point-of-Care-Tests in Deutschland, kategorisiert nach Erregern und Methodik

Erreger	Methodik	Testname	Hersteller
HIV	LFA	autotest VIH®	AAZ-LMB, Boulogne-Billancourt, Frankreich; Vertrieb: ratiopharm, Ulm, Deutschland
		Biosure HIV Self Test	Biosure, Grass Valley, California, USA
		Exacto Pro HIV	Biosynex, Illkirch-Graffenstaden, Frankreich
		INSTI HIV Self-Test	bioLytical, Richmond, British Columbia, Canada
		OraQuick HIV-Selbsttest	OraSure Technologies, Inc., Bethlehem, Pennsylvania, USA
		Simplitude ByMe HIV-Selbsttest	Atomo Diagnostics, Ltd., Leichhardt, New South Wales, Australien; Vertrieb: Owen Mumford, Großostheim-Ringheim, Deutschland
		Determine™ HIV Early Detect (m. p24-Antigen-Detektion)	Abbott Diagnostics, Wiesbaden, Deutschland
Hepatitis C	LFA	Bioline™ HCV	Abbott Diagnostics, Wiesbaden, Deutschland
		OraQuick® HCV Rapid Antibody Test	OraSure Technologies, Inc., Bethlehem, Pennsylvania, USA
		Rapid Anti-HCV Test	InTec Products, Xiamen, Fujiang, Volksrepublik China
		STANDARD™ Q HCV Ab Test	SD Biosensor, Giheung-gu, Korea
	PoC-NAT	Xpert® HCV Viral Load	Cepheid, Sunnyvale, Kalifornien, USA
		GeneXpert® HCV VL Fingerstick	Cepheid, Sunnyvale, Kalifornien, USA
		Genedrive® HCV	Genedrive, Manchester, Großbritannien
		Molbio Truenat® HCV	Molbio Diagnostics, Ltd., Goa, Indien
		SAMBA II HCV Qual	DRW, Diagnostics for thr Real World, Morgan Hill, Kalifornien, USA
Syphilis	LFA	Determine™ Syphilis TP	Abbot Diagnostics, Wiesbaden, Deutschland
NG/CT	PoC-NAT	GeneXpert-CT/NG-Assay	Cepheid, Sunnyvale, Kalifornien, USA
		Binx io CT/NG-Duplex-Test	Binx health, Cambridge, Massachusetts, USA
	RT-PCR	Sexual Health Click Test	Visby Medical, San Jose, Kalifornien, USA
CT	LFA	aQcare Chlamydia TRF Test	Medisensor, Daegu, Südkorea
NG/CT/MG	PoC-NAT	Cobas® Liat ® System^a^	Roche, Basel, Schweiz

^a^ Liat-Assays im Testformat CT/NG/MG sind voraussichtlich ab Mitte 2025 verfügbar

*CT Chlamydia trachomatis, LFA* Lateral Flow Assay, *MG Mycoplasma genitalium, NG Neisseria gonorrhoeae, PoC-NAT* Point-of-Care-Nukleinsäureamplifikationstests, *RT-PCR* reverse Transkriptase-Polymerase-Kettenreaktion

Eine Herausforderung in der HIV-Diagnostik stellt die sogenannte diagnostische Lücke dar, der Zeitraum zwischen der stattgefundenen Ansteckung bis zur Serokonversion (Nachweis von HIV-spezifischen Antikörpern), der im Bereich von 2–8 Wochen liegen kann. Daher beruhen modernere Labortestsysteme auf einer Antigenkomponente mit Nachweis des p24-Kapsidproteins in Kombination mit der Detektion von Antikörpern, sodass mit den konventionellen laborbasierten Verfahren eine Infektion früher (häufig bereits 6 Wochen nach Exposition) mit hoher Wahrscheinlichkeit ausgeschlossen werden kann [27]. Alle in Deutschland verfügbaren HIV-Selbsttests detektieren nur die Antikörperkomponente, weswegen ein negatives Ergebnis erst 12 Wochen nach Exposition aussagekräftig ist. Als Kombinationstest mit zusätzlicher p24-Antigendetektion ist der Determine™ HIV Early Detect in Deutschland derzeit zwar als PoCT, aber nicht als Selbsttest zugelassen. Die Detektion der Antigenkomponente kann die diagnostische Lücke im Frühstadium der HIV-1-Infektion häufig verkürzen, jedoch ist die Sensitivität der p24-Detektion nichtlaborbasierter Verfahren insgesamt eher niedrig. In einer unabhängigen Testung konnte dieser aktuelle Kombinationstest 77 % der Plasmaproben von Patientinnen und Patienten mit akuter HIV-1-Infektion im Fiebig-Stadium II-III, also Immunoblot-negative Probe, korrekt als positiv erkennen, wohingegen die Vorgängerversion nur 25 % erkannte [28]. Die klinische Sensitivität der PoCT ist jedoch teils niedriger als die unter Laborbedingungen durchgeführte Testung der analytischen Sensitivität. Dies liegt u. a. daran, dass bei den allermeisten laborbasierten Studien Plasma- oder Serumproben untersucht werden, wohingegen in der klinischen Anwendung Vollblut eingesetzt wird. Alle verfügbaren Selbsttests basieren auf der Detektion von Antikörpern aus Kapillarblut, mit Ausnahme des OraQuick®-HIV-Selbsttest, bei dem Mundflüssigkeit eingesetzt wird. Die Sensitivität des OraQuick®-Tests schnitt in manchen Untersuchungen jedoch im Vergleich zu den auf Blut basierenden Selbsttestverfahren mit bis zu 66,7 % schlechter ab [26].

Durch die Einnahme einer medikamentösen Präexpositionsprophylaxe (PrEP) kann im Falle einer HIV-Infektion die Serokonversion verzögert und die diagnostische Lücke vergrößert werden. So betrug in einer retrospektiven Analyse die durchschnittliche Zeitspanne bis zur Reaktivität im Immunoblot (Fiebig-Stadium V) bei Patientinnen und Patienten unter PrEP 28 Tage gegenüber 17 Tagen bei Patientinnen und Patienten ohne PrEP [29]. Die verzögerte Serokonversion beruht vermutlich auf der Reduktion der HIV-Antigenlast durch die antiretroviralen Substanzen der PrEP. HIV-PoCT bzw. Selbsttests sind somit nur bedingt für die Diagnostik bei PrEP-Anwendenden einsetzbar und sollten nicht unter antiretroviraler Therapie (ART) verwendet werden, da hier falsch-negative Ergebnisse deutlich häufiger auftreten. In diesen Konstellationen sollten vorzugsweise laborbasierte Verfahren zum Einsatz kommen und gegebenenfalls Verlaufstestungen durchgeführt werden.

## Hepatitis C

Die Leitlinien zur Diagnostik einer HCV-Infektion sehen ein Stufenschema vor, in dem zunächst auf HCV-Antikörper untersucht wird [30]. Ein reaktives Ergebnis weist auf eine HCV-Infektion hin, wobei zwischen einer ausgeheilten und einer replikativen Infektion nicht unterschieden werden kann. Dazu ist ein direkter Virusnachweis in der Regel durch Bestimmung der HCV-RNA notwendig. Für beide Schritte der Stufendiagnostik stehen verlässliche Testsysteme zur Verfügung, die patientennah durchgeführt werden können. Hier werden die Systeme erwähnt, die von der WHO präqualifiziert wurden.

Für den Nachweis von HCV-Antikörpern werden derzeit durch die WHO 4 immunchromatografische PoCT als präqualifiziert gelistet (Tab. 1). Laut WHO liegen die Sensitivitäten der Tests bei 99,3–100 %, die Spezifitäten bei 99,4–100 % [31]. Eine unabhängige Studie bestätigt die hohe Zuverlässigkeit (mittlere Sensitivität: 94,15–96,1 % und mittlere Spezifität 99,15–99,95 %; [32]). Neben der Antikörperbestimmung aus Blut ist das System OraQuick® auch für die Untersuchung aus einem Abstrich der Mundschleimhaut zugelassen (Sensitivität 89,9 %; Spezifität 100 %; [33]). Die verfügbaren immunchromatografischen PoCT für den Nachweis von HCV-Antikörpern sind damit weitgehend zuverlässig. Allerdings erlauben die Testergebnisse keine Aussage zur Infektiosität oder Behandlungsindikation. Da etwa ein Drittel der akuten HCV-Infektionen spontan ausheilt und in den letzten Jahren der Anteil der erfolgreich behandelten chronischen HCV-Infektionen deutlich zugenommen hat, hat auch in Risikogruppen der prädiktive Wert eines reaktiven Anti-HCV-Tests für den Nachweis von Infektiosität abgenommen. Der direkte Virusnachweis ist deshalb der entscheidende Parameter.

Dieser erfolgt üblicherweise durch eine Untersuchung der HCV-RNA. Obwohl Tests für den Nachweis des HCV-coreAg für serologische Vollautomaten verfügbar sind, stehen keine verlässlichen PoCT für den patientennahen Antigennachweis zur Verfügung. Für den Nachweis und die Quantifizierung der HCV-RNA ist als PoC-NAT das System GeneXpert von Cepheid zugelassen [31]. Der Test liefert innerhalb von etwa 60–100 min verlässliche Ergebnisse für die HCV-Genotypen 1 bis 6 (Limit of Detection 5 IE/ml bzw. 60 IE/ml aus Kapillarblut der Fingerbeere; [31]). Weitere qualitative und quantitative Testsysteme sind zum Nachweis der HCV-RNA als PoC-NAT mit einer etwas höheren Nachweisgrenze zugelassen (Tab. 1).

## Syphilis

Es gibt wenig Neuerungen im Bereich der PoCT für die Syphilisdiagnostik. Für einige auf dem Markt verfügbare PoCT wie den Determine™ Syphilis TP werden eine sehr gute Sensitivität (100 %) und Spezifität (100 %) angegeben. Diese Tests beruhen meist auf dem Nachweis spezifischer Syphilisantikörper. Im Rahmen einer Präqualifizierungsprüfung nach IVDR der WHO lag die Spezifität für den genannten Test mit 98,7 % etwas niedriger als laut Herstellerangaben [34]. Eine Nachtestung ist allerdings auch bei positiven Ergebnissen immer erforderlich. Der PoCT kann als Screeningtest eingesetzt werden, gibt aber keine Information zu der Krankheitsaktivität. Ein positives Ergebnis im PoCT kann demnach nicht zwischen einer lange ausgeheilten Syphilisinfektion (sog. Seronarbe) und einer aktiven Syphilisinfektion unterscheiden. Als Alternative zur PoCT-Diagnostik bietet sich daher für die Syphilisdiagnostik eine Analyse aus Trockenblut an, die eine vergleichbare Niedrigschwelligkeit ermöglicht. Hierbei kann ein Screening über einen Enzymimmunoassay(EIA-)Test sowie eine Bestätigungs- und Aktivitätsdiagnostik über den VDRL/Treponemen-IgM-Immunblot durchgeführt werden und eine Differenzierung zwischen aktiven und ausgeheilten Infektionen erfolgen.

## Gonokokken

Ein korrekt vorbereiteter, nach Gram gefärbter und mikroskopisch untersuchter Abstrich zur Identifizierung gramnegativer intrazellulärer Diplokokken in polymorphkernigen Leukozyten ist empfindlich (83–95 %) und spezifisch (92–99 %) für die Diagnose der Gonorrhö bei symptomatischen Männern mit Ausfluss aus der Harnröhre [35, 36] und kann bei entsprechenden apparativen Voraussetzungen und erfahrenem Untersuchenden als PoCT eingesetzt werden. Eine Methylenblaufärbung kann alternativ verwendet werden, ist aber etwas weniger spezifisch. Wegen geringer Sensitivität eignet sich die Direktmikroskopie jedoch nicht für den Ausschluss einer urethralen Infektion bei asymptomatischen Männern oder einer endozervikalen, pharyngealen oder rektalen Infektion.

Die aktuell verfügbaren antigenbasierten Schnelltests sind nicht geeignet, um eine NG-Infektion zuverlässig nachzuweisen, da sie nur über eine unzureichende diagnostische Genauigkeit verfügen. [37–41]. Die in klinischen Evaluationen von Antigen-PoCT berechnete Sensitivität und Spezifität lagen zwischen 12,5 % und 94 %, bzw. 89 % und 99,8 % [38]. Die in einigen Studien angegebene relativ hohe Sensitivität und Spezifität müssen jedoch mit Vorsicht interpretiert werden, da das jeweilige Testsetting nicht immer die reale Prävalenz von NG-Infektionen abbilden konnte, die Probenanzahl zu klein war und zudem ein suboptimaler Referenztest verwendet wurde, sodass die klinische Sensitivität des Antigenschnelltests deutlich niedriger sein dürfte [41, 42]. Weiterführende technologische Entwicklungen im Bereich Mikrofluidik und Biosensoren konzentrieren sich auf das Multiplexing-Potenzial von Immunoassay-Tests und den parallelen Nachweis von CT und NG in Urinproben, befinden sich aber noch in einem frühen Stadium [15, 43].

NAT stellen den Goldstandard in der NG-Diagnostik dar [39], da im Vergleich zur Kultur keine vitalen Organismen benötigt werden, was die Entnahme und Verwendung vielfältigerer biologischer Proben ermöglicht. NAT-Diagnostik kann auch zum Nachweis extragenitaler NG-Infektionen (rektal, pharyngeal) genutzt werden [44]. NAT haben eine hohe Sensitivität, auch beim Nachweis asymptomatischer Infektionen mit geringer Organismenlast [15]. Als vollautomatisierter PoC-NAT für NG ist der GeneXpert-CT/NG-Assay verfügbar. In der Analyse von Erststrahlurinproben von Männern sowie vaginalen und zervikalen Abstrichproben konnte der Test NG-Infektionen mit hoher Sensitivität (97,4–98,7 % bzw. 98–100 %) und Spezifität (99,4–99,9 % bzw. 99,9–100 %) nachweisen und unterscheidet sich damit nicht von Standard-Labor-NAT [41, 45]. Die Testergebnisse liegen nach 90 min vor. Im April 2019 wurde die FDA-Zulassung des GeneXpert-CT/NG-Assays auf extragenitale Proben (rektale- und oropharyngeale Abstriche) erweitert, da für rektale Abstrichproben keine signifikanten Unterschiede zwischen den Assays gefunden wurden [46, 47]. In pharyngealen Abstrichproben war die Sensitivität des GeneXpert mit 77,8 % dagegen niedriger [48]. Bei der Verwendung gepoolter Proben, insbesondere von Urinproben, für den Nachweis von NG mit GeneXpert wurde eine verringerte Empfindlichkeit beobachtet, ähnlich den Ergebnissen, die in laborgestützten NG-Pooling-Studien berichtet wurden [49].

Eine klinische Multicenterstudie mit 1500 Patientinnen und Patienten zeigte für den Binx-io-CT/NG-Duplex-Test bei vaginalen Abstrichen eine Sensitivität von 100 % und eine Spezifität von 99,9 %. Bei Urinproben von Männern lagen die Sensitivität und Spezifität für NG bei 97,3 % bzw. 100 % und damit vergleichbar mit GeneXpert. Die Ergebnisse liegen nach bereits 30 min vor [50].

Der Sexual-Health-Click-Test auf der Basis einer RT-PCR zeigte in einer „cross-sectional, single-visit study“ mit 1486 Patientinnen überzeugende Ergebnisse in der Multiplexdiagnostik mit einer Sensitivität von 97,4 % (86,5–99,5 %) und Spezifität von 99,4 % (98,9–99,7 %) für den Nachweis von NG. Der instrumentenfreie Test wurde gegen das Hologic Aptima Combo 2 Assay und das BD ProbeTec CT/GC Assay getestet. Das Testergebnis lag in unter 30 min vor [51]. Mehrere weitere Plattformen mit unterschiedlichen Assays und Verwendung verschiedener Amplifikationstechnologien sind in der Entwicklung.

Eine besondere Herausforderung stellt die weltweit zunehmende Resistenzentwicklung bei NG dar. PoC-NAT für NG liefern jedoch keine Informationen zur Antibiotikaresistenz [52]. Eine Möglichkeit, PoC-NAT zu nutzen, liegt darin, einen Abstrich zur kulturellen Diagnostik anzuschließen, nachdem ein positiver PoCT vorliegt. Resistenzassoziierte molekulare Veränderungen sind bisher nur teilweise gut charakterisiert und eine Reihe von Methoden wurden publiziert, mit denen einzelne oder mehrere Resistenzdeterminanten detektiert werden können [53–55]. Eine molekulare Empfindlichkeitstestung kann jedoch eine kulturelle Empfindlichkeitstestung derzeit nicht ersetzen, insbesondere im Hinblick auf das *First-Line*-Therapeutikum Ceftriaxon.

## Chlamydien

Die meisten antigenbasierten Tests für CT erreichen nach wie vor die Vorgaben [14] an PoCT-Formate nicht hinreichend. In einer Cochrane-Analyse (19 Studien, 13.676 Datensätze) zeigten die 9 untersuchten Assays zwar eine relativ hohe Spezifität (98 %), aber nur eine gepoolte Sensitivität von 48 % (endozervikal: 48 %, Urin/vaginal: 49 %; [56]). In einer zweiten Übersichtsarbeit (39 Studien, 42.336 Datensätze) werden 10 antigen- und 4 NAAT-basierte PoCT bewertet [57] und auch hier unterschreiten die meisten Antigen-PoCT mit 51,6 % die Zielvorgabe zur Sensitivität (mindestens 90 %) erheblich, zeigten aber eine gute Spezifität von 98,8 %. Zervikale und vaginale Abstriche rangieren auf identischem Sensitivitätsniveau (51,6 % vs. 54,0 %). Für Urin ergeben sich heterogene Ergebnisse (Sensitivitäten zwischen 45,4 % und 88,2 %). Wurde Erststrahlurin verwendet, erhöht sich die Sensitivität auf über 80 %. Unter allen untersuchten antigenbasierten Assays erreicht alleine der aQcare-Chlamydia-TRF-Test aufgrund seiner Nanopartikeltechnologie die angestrebten Performancegrenzen (Spezifität: 95,8 % Sensitivität: 91,0 %).

Im Gegensatz zu antigenbasierten PoCT zeigten alle untersuchten NAT-Assays eine gute gepoolte Sensitivität von 95,5 % bei identischer Performance für zervikale (95,7 %) und vaginale Proben (96,1 %). Die Spezifität betrug gepoolt 99,4 %. Da PoC-NAT mittlerweile sehr akkurat detektieren, spielen Unterschiede in der technischen Handhabung, den Kosten und insbesondere in der Testdauer eine Rolle, so benötigt z. B. der Xpert ca. 80–90 min, das io-System 30 min und das Liat-System 20 min bis zur Ergebnismitteilung (Liat-Assays im Testformat CT/NG/MG sind voraussichtlich ab Mitte 2025 verfügbar; Tab. 1).

Mit Ausnahme des aQcare-Chlamydia-TRF-Test sind antigenbasierte Testsysteme zur PoC-Diagnostik nicht zu empfehlen und stattdessen PoC-NAT zu bevorzugen [57, 58].

## Mykoplasmen

Unter den zellwandlosen Spezies der Ordnung Mycoplasmatales sind *Mycoplasma genitalium *(MG), *M. hominis, Ureaplasma parvum* und *U. urealyticum* sexuell übertragbar. Während MG als Pathogen inzwischen etabliert ist, werden die übrigen Arten nach gegenwärtigem Kenntnisstand als Kommensalen angesehen, die unter bestimmten Bedingungen Auslöser von Symptomen urogenitaler Infektionen sein können. Für die Diagnostik bleibt die Kultivierung von MG weiterhin außerordentlich schwierig, sodass im Routinelabor ausschließlich NAT für die Detektion des Erregers zum Einsatz kommen. Dagegen sind Ureaplasmen und M. hominis über Spezialmedien in der Regel nach 24–48 h kulturell nachzuweisen. Darüber hinaus sind auch für diese Organismen RT-PCR-Tests zur schnellen und spezifischen Bestätigung einer Kolonisation verfügbar, die z. T. die Differenzierung der beiden Ureaplasmaarten gestatten. Therapieausfälle mit persistenten Beschwerden sollten insbesondere bei MG, aber auch bei den übrigen Spezies Anlass für eine Resistenztestung sein. Für diesen Erreger stehen dazu inzwischen molekulare Tests zur Verfügung, während die Empfindlichkeit von *M. hominis* und *Ureaplasma spec*. vorrangig phänotypisch bestimmt wird. Die nicht abschließend geklärte Epidemiologie der Infektionen durch Mykoplasmen, ihr Vorkommen bei einer großen Zahl asymptomatischer Personen und der für praktische Fragestellungen offensichtlich ausreichend schnelle Nachweis mit den derzeit verfügbaren Verfahren dürften wesentliche Ursachen dafür sein, dass PoCT bisher keinen Eingang in die Labore gefunden haben. Obwohl entsprechende, meist auf der Loop-mediated-Isothermal-Amplification-(LAMP-)Technologie basierende Ansätze in der Literatur beschrieben sind [59], verhindert nicht zuletzt die verlässliche Validierung dieser Alternativen ihre kommerzielle Nutzung und damit den breiten Einsatz in der gegenwärtigen Praxis.

## Meldung positiver PoCT

Ob positive PoCT auf STI gemäß IfSG gemeldet werden müssen, hängt von den für jeden Erreger spezifischen Falldefinitionen des RKI ab. Für Infektionen mit CT und MG existiert in Deutschland keine Meldepflicht. Für Erreger, für die gemäß IfSG eine Meldepflicht besteht, sind die jeweiligen Bedingungen wie folgt:

### HCV.

Gemäß § 7 Abs. 1 Satz 1 Nr. 22 IfSG besteht eine namentliche Meldepflicht für alle direkten und indirekten Nachweise von HCV, unabhängig vom klinischen Bild und Infektionsstadium, insofern auch für PoCT. Die Meldepflicht entfällt, wenn die Meldung bereits erfolgte.

### HIV.

Die aktuell verfügbaren HIV-PoCT entsprechen in der Aussagekraft den Suchtests zur Antikörpererkennung der klassischen Labordiagnostik. Da eine Bestätigung durch einen Immunoblot bzw. ein direkter Erregernachweis durch eine NAT nicht gegeben ist, fehlt ein vollständiger direkter oder indirekter Nachweis einer HIV-Infektion gemäß § 7 Abs. 3 IfSG. Ein positiver HIV-PoCT ist daher nicht meldepflichtig.

### Gonokokken.

NG-Infektionen sind nach § 7 Abs. 3 IfSG meldepflichtig. Alle bisher verfügbaren NG-PoCT können eine Infektion allerdings nicht sicher nachweisen, daher sind positive PoCT-Ergebnisse nicht meldepflichtig.

### Syphilis.

Gemäß § 7 Abs. 3 IfSG ist der direkte oder indirekte Nachweis einer Syphilisinfektion zu melden. Zur Bestimmung einer aktiven Infektion müssen geeignete Parameter zur Bestimmung der Behandlungsbedürftigkeit (z. B. IgM- oder Lipoidantikörper oder positives PCR-Ergebnis) bestimmt werden, da sich die Behandlungsbedürftigkeit nicht aus den bisher verfügbaren PoCT ableiten lässt, eine Meldung ist daher nicht nötig. Ein positives PoCT-Ergebnis ist durch eine reguläre Stufendiagnostik abzusichern und zu ergänzen.

## Diskussion

Für den Einsatz in niedrigschwelligen Einrichtungen stehen PoCT für HIV, HCV und Syphilis mit überzeugender Qualität zur Verfügung. Für HIV und Syphilis sind diese auch als Selbsttest zugelassen. Für die Detektion anderer Infektionen, wie z. B. CT, gibt es einzelne vielversprechende Entwicklungen, die sich in der Praxis jedoch noch beweisen müssen. Für die Detektion von NG und MG gibt es aufgrund mangelnder Testgüte bisher keine PoCT, die für einen Einsatz in niedrigschwelligen Einrichtungen oder als Selbsttest geeignet wären.

Die vermehrte Anwendung von PoCT, inkl. PoC-NAT, etwa in niedrigschwelligen Einrichtungen hat Vorteile für die klinische Praxis. Durch deren Anwendung können Infektionskrankheiten schneller und häufiger erkannt und zielgerichtet behandelt werden. Dies ist wichtig vor allem für Personen, die einen erschwerten Zugang zu Beratungs‑, Test- und Behandlungsangeboten haben, da häufig nur ein einmaliger Kontakt mit einer Einrichtung des Gesundheitswesens stattfindet. Hierdurch können der Zugang zur Behandlung und die Therapieadhärenz verbessert und die Übertragung von STI deutlich reduziert werden [60–62]. Auch für die HCV-PoC-NAT und die konsekutive Behandlung von HCV konnte der deutliche Vorteil niedrigschwelliger Angebote auf Behandlungsraten, Behandlungserfolg, Anzahl der Reinfektionen sowie Lost-to-Follow-up im Vergleich zu niedrigschwelliger Testung und Behandlungsangebot im Krankenhaus gezeigt werden [9]. Entsprechend werden niedrigschwellige HCV-Test-and-Treat-Angebote mit PoCT für Drogengebrauchende vom Europäischen Zentrum für die Prävention und die Kontrolle von Krankheiten (ECDC) im Rahmen der Eliminierungsstrategie empfohlen [63].

Gleichzeitig könnten durch die vermehrte Anwendung von PoCT Nachteile entstehen. Es ist wahrscheinlich, dass nicht alle Personen mit einem positiven PoCT-Ergebnis eine ärztliche Konsultation und Folgediagnostik in Anspruch nehmen werden, weswegen manche Infektionen trotz einer vorläufigen Diagnose unbehandelt bleiben. Eine Folgediagnostik und Überführung in eine Behandlung gelingen jedoch auch nicht immer bei einer labordiagnostischen Testung.

Bei einem niedrigen positiven Vorhersagewert eines PoCT kann es durch falsch-positive Testergebnisse auf individueller Ebene zu unnötigen psychischen Belastungen sowie zu einer Fehl- und Übertherapie und auf kollektiver Ebene zu einer vermehrten Belastung des Gesundheitssystems (Rapid Test Paradox) kommen. Dagegen werden bei einem unbefriedigend niedrigen negativen Vorhersagewert nicht alle Infektionen erkannt, woraus sowohl individuelle klinische Nachteile als auch die weitere Verbreitung nichtdiagnostizierter STI erwachsen können. Bei der Anwendung von PoCT jeder Art in der medizinischen Versorgung muss eine stetige Qualitätssicherung erfolgen. So sollten etwa Programme zur Schulung der Anwendenden, hausinternen Validierung, Nutzung von Kontrollen (Kontrollstämme und -materialien) sowie Ringversuche konzipiert werden [64]. Zuletzt entstehen durch die Nutzung von PoC-NAT potenziell höhere finanzielle Kosten für die Krankenkassen oder – bei nicht gegebener Erstattung – für die Patientinnen und Patienten.

## Fazit

Der Einsatz von PoCT und Selbsttests für HIV, HCV und Syphilis in Deutschland kann niedrigschwellige Testangebote unterstützen und somit die Möglichkeiten einer früheren Diagnosestellung verbessern. Für andere STI gilt dies noch nicht.

Die IVD-Verordnung gilt seit dem 26.05.2022. Sie wird in einer gestaffelten Übergangszeit umgesetzt, vom 26.05.2025 für in Klasse D, also mit dem höchsten Risiko gelistete STI (HIV, HBV, HCV) bis zum 26.05.2026 für andere STI (HSV, HPV, NG, Syphilis, CT usw.), die in Klasse C gelistet sind, und bis zum 26.05.2028 für Inhousetests. Dies bedeutet, dass viele STI-Tests, die bisher von den Herstellern unter der alten IVD-Richtlinie selbst deklariert werden konnten, einer Bewertung durch benannte Stellen unterzogen werden, womit ein höheres Qualitätsniveau zu erwarten ist. Neuere Entwicklungen im Bereich von Amplifikationstechnologien werden die PoC-NAT-Diagnostik in Zukunft ebenfalls nachhaltig beeinflussen.

Es besteht die Hoffnung, dass die oben genannten technischen und regulatorischen Entwicklungen die langfristige Integration der PoC für STI in Deutschland befördern und hochqualitative niedrigschwellige Testangebote, wie von der WHO verlangt, verstärken werden [65].
